# Supplementary material for: ChatGPT as a Tool for Biostatisticians: A Tutorial on Applications, Opportunities, and Limitations
Source: Stat Med. 2025 Oct 23;44(23-24):e70263. doi: 10.1002/sim.70263 (PMC12548020; doi:10.1002/sim.70263)
Supplement: Supplementary file 1 — Data S1. Supporting Information. [file SIM-44-0-s001.zip › Supplementary Materials Additional.zip!/3.1_Systematic_Reviews_and_Meta_aspects/3.1_Data Extraction - o4-mini - Chat 3.pdf]

OpenAI

Use Case Meta-Analysis - Data Extraction - o4-mini - Chat 3

Rudloff et al. - 2009 - Concurrent lobular neoplasia increases the risk of ipsilateral breast cancer recurrence in situ treated with breast-conserving therapy.pdf
PDF

Vidali et al. - 2012 - Conservative treatment of breast ductal carcinoma in situ results of an Italian multi-institutional.pdf
PDF

The aim is to perform a meta-analysis on total ipsilateral local recurrence rates at 10 years in cases of ductal carcinoma in situ.

Data inclusion rules are: To ensure the validity of the follow-up data, the results should be as close to 10 years follow-up (minimum) as the information in each study allows. If there is more than one publication from an institution or group, the most recent study with appropriate follow-up will be used to extract 10-year data.

Please provide from each of the two attached studies:

- the rate of ipsilateral local recurrence for conservative surgery with additional radiotherapy (no other interventions or drugs allowed) after 10 years

- the number of eligible patients

- period of data collection

- duration of follow-up

- type of study

- age of patients (mean or median, maximum and minimum)

- treatment modalities

For this task you have access to up-to-the-minute web retrieval to find adequate approaches for data extraction. When answering the task above please proceed as follows:

1. Fetch the most relevant sources.

2. Show your step-by-step reasoning.

3. At the end, give me a concise answer citing the sources you used.

4. Please provide a dataframe with one entry for each study, and column names "study", "rate", "n_patients", "period", "follow_up", "study_type", "age", and "treatment".

Meta-analysis Data Extraction

	study	rate	n_patients	period
1	Rudloff et al. (2009)	11.9% (10-year IBTR with surgery + RT)	91	1991–1995
2	Vidali et al. (2012)	9.6% (10-year actuarial local recurrence)	586	Feb 1985–Mar 2000

Here's the concise answer with the requested details and citations:

Rudloff et al. (2009)

- **Rate (10-year ipsilateral recurrence)**: 11.9% for patients receiving breast-conserving surgery + external-beam radiotherapy
- **Number of eligible patients**: 91 (those treated with RT)
- **Period of data collection**: 1991–1995
- **Duration of follow-up**: Median 11 years (range 0–16 years)
- **Type of study**: Retrospective institutional cohort review
- **Age of patients**: Median 55 years (range 26–89 years)
- **Treatment modalities**: Breast-conserving surgery plus external-beam radiotherapy; no chemotherapy and only 6% received tamoxifen (excluded per inclusion rule)

Vidali et al. (2012)

- **Rate (10-year ipsilateral recurrence)**: 9.6% (actuarial local recurrence)
- **Number of eligible patients**: 586
- **Period of data collection**: February 1985–March 2000
- **Duration of follow-up**: Median 136 months (range 16–292 months)
- **Type of study**: Retrospective multi-institutional study
- **Age of patients**: Median 55 years (range 29–84 years)

- **Treatment modalities**: Conservative surgery followed by whole-breast radiotherapy (median total dose 50 Gy; 50% received a 10 Gy boost)

The interactive table above summarizes these key data points for your meta-analysis.

Quellen
